# MRI T2 Hypointensities in basal ganglia of premanifest Huntington's disease

**DOI:** 10.1371/currents.RRN1173

**Published:** 2010-09-08

**Authors:** Caroline K Jurgens, Radu Jasinschi, Ahmet Ekin, Marie-Noëlle W Witjes-Ané, Jeroen van der Grond, Huub Middelkoop, Raymund A.C. Roos

**Affiliations:** ^*^Leiden University Medical Center, Leiden; Bronovo hospital, The Hague, The Netherlands; ^†^Philips Research, Eindhoven; ^‡^Philips Research; ^¶^LUMC; ^#^Leiden University Medical Center and ^**^LUMC, Leiden, The Netherlands

## Abstract

Increased iron levels have been demonstrated in the basal ganglia of manifest Huntington’s disease (HD). An excess in iron accumulation correlates with MRI T2-weighted hypointensity. Determination of the amount of hypointensities in the basal ganglia in the premanifest phase of HD may give more insight in the role of iron in the pathogenesis of HD. Therefore, the present study assessed whether the degree of hypointensities on T2-w MRI in the basal ganglia of premanifest gene carriers differs from non-carriers. Seventeen HD gene carriers without clinical motor signs and 15 non-carriers underwent clinical evaluation and MRI scanning. The amount of T2-w hypointensities was determined using a computer-assisted quantitative method that classified each pixel in the basal ganglia as hypointense or not, resulting in a total of hypointense pixels for each individual. Carriers showed an increased amount of hypointensities in the basal ganglia compared to non-carriers. More hypointensities were furthermore associated with a higher UHDRS total motor score, a longer CAG repeat length and a greater probability of developing symptoms within 5 years. We concluded that the increased amount of hypointensities in the basal ganglia of premanifest carriers of the HD gene may reflect excessive iron deposition and a role for iron in the neuropathology of HD. Furthermore, this phenomenon is associated with clinical and biological disease characteristics. An increased amount of hypointensities on T2-w MRI in the basal ganglia may be considered a biomarker for HD.

## Introduction

Huntington’s disease (HD) is an inherited neurodegenerative disorder that becomes manifest in midlife and is characterized by progressive motor, cognitive and behavioral dysfunction [1]. The genetic defect, a CAG repeat expansion, results in a malformed huntingtin protein that initiates events leading to neuronal loss especially in the basal ganglia, the most iron rich area of the brain. There is increasing evidence that iron is involved in the mechanisms that underlie many neurodegenerative diseases. Excessive and premature deposition of iron in the basal ganglia has been reported in neurodegenerative diseases like Alzheimer’s disease and Parkinson’s disease [2] [3] [4]. The role of iron in the pathogenesis of HD remains unknown. Increased iron levels in the brain of Huntington’s disease patients have been demonstrated post-mortem [5] [6] [7]. Brain iron can also be measured indirectly by MRI, through its effect on transverse relaxation times (T2) [8] [9] [10]. Increased T2-weighted hypointensity (i.e. decreased signal intensity) has been linked to high levels of iron accumulation [11] [12] [13]. In HD patients altered T2 relaxation times in the basal ganglia have been demonstrated even early in the disease, and were attributed to pathological iron deposition [10] [14] [15] [16]. Up to now possible iron accumulation has not been studied in carriers of the HD gene who are still without clinical signs. Therefore, this is the first study to examine signal hypointensities in the premanifest phase of HD and may give more insight in the role of iron in the earliest pathological brain changes related to HD.The aims of the present explorative study were; (1) to assess whether the degree of hypointensities on T2-w MRI in the basal ganglia of premanifest carriers of the HD gene differs from non-carriers, (2) to assess the possible association between the amount of hypointensities and clinical and biological measures. 

## Materials and methods

Seventeen premanifest gene carriers (further labelled as carriers) and 15 non-carriers participated in this study. All participants were recruited from the Leiden University Medical Centre (LUMC) outpatient Neurological department. Participants had undergone gene testing according to international guidelines at an earlier time [17]. The median CAG repeat length in carriers was 42 (range 40-49) and in non-carriers 19 (range 16-24). The estimated probability of symptom onset within 5 years was determined [18]. Carriers were considered ‘premanifest’ in the absence of ‘definite’ motor signs on the Unified Huntington’s Disease Rating Scale (UHDRS) [19], as assessed during their last visit to our outpatient department. Reassessment of motor functioning during study enrolment by a neurologist blind to genetic status, resulted in the exclusion of one carrier who was rated as definite HD. One non-gene carrier who showed evidence of overt cerebral damage on MRI was also excluded from analyzes. Another non-carrier was excluded because of unsatisfactory quality of the T2-w scan. Therefore, analyzes were performed on 16 carriers and 13 non-carriers.The study had been approved by the local Medical Ethical Committee. Written informed consent was obtained from all participants.

### Procedure

All participants were evaluated with the UHDRS and MRI of the brain. All MRI evaluations were performed within 4 months after clinical evaluation (mean: 30 days, SD: 44 days).From the motor part of the UHDRS the Total Motor Score (TMS) was used (range 0-124), with higher scores representing more motor abnormalities. The cognitive and behavioral sections of the UHDRS were administered by a psychologist. The Total Behavioral Score was obtained by adding the products of frequency and severity for each item [20]. 

### Image acquisition

All imaging was performed on a whole body MR system operating at field strength of 3.0 Tesla (Philips Medical Systems, Best, The Netherlands). MRI consisted of a 3D-T1-weighted and T2-weighted scan. Acquisition parameters were as follows: 3D-T1-weighted: TR = 9.8 msec; TE = 4.6 msec; flip angle = 8°; section thickness = 1.2 mm; number of sections = 120; no section gap; whole brain coverage; FOV = 224 mm; matrix = 192, reconstruction matrix = 256; T2-weighted: TR = 3951 msec; TE = 80 msec; flip angle = 90°; section thickness = 3.6 mm; number of sections = 40; no section gap; whole brain coverage; FOV = 224 mm; matrix = 448, reconstruction matrix = 1024, Turbo-Spin-Echo factor = 16.

### Image postprocessing

Image postprocessing was performed through collaboration between the Department of Radiology from the LUMC and Philips Research Eindhoven. First images were registered to the Talairach-Teroux brain atlas, to define the Region of Interest (ROI) (basal ganglia) as a subset of grid cells [21]. Three adjacent slices in each individual were analyzed covering the entire basal ganglia. The signal intensity of each tissue pixel of the basal ganglia cells (Fig 1, red cells) was scaled according to that of reference tissue (Fig 1, green cells). This indicates the deviation from normality in tissue appearance. Subsequently, the resulting signal intensities were normalized by dividing this difference by the (average) image brightness of the reference cells. This is reflected in the following formula where *HypoMR (x,y)* denotes the normalized value and *I (x,y)* the basal ganglia pixel intensities, and *M_ref_* denotes the average brightness in the reference cells;

**Figure:**
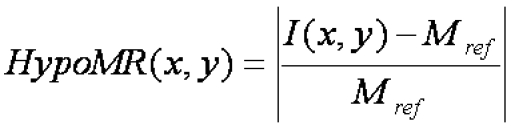

**Figure 1. Basal ganglia cells (ROI) (in red) and reference cells (in green). Hypointense pixels are displayed in yellow/orange (example of one gene carrier)**

**Figure:**
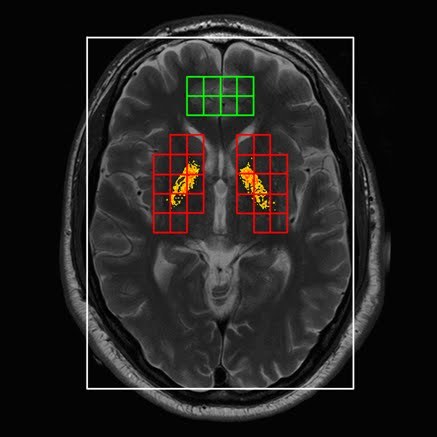

Our criteria to detect a hypointense pixel in the normalized T2-w image are based on the use of a single threshold. The threshold was determined using qualitative judgements of each pixel in 20 control scans derived from the LUMC. Two radiological specialists classified each pixel as either hypointense or not. Based on these findings the optimal threshold λ for this technique was estimated. Each pixel in the ROI in our study was automatically scored as hypointense if *HypoMR (x,y)*<λ. The hypointense pixels in each slice were highlighted in yellow/orange (Fig 1). This results in the total amount of hypointensities in the basal ganglia for each individual and for the left and right hemisphere separately. 

Brain volumes were derived from 3D-T1-w scans. Whole brain volume was automatically determined using the cross-sectional version of the Structural Image Evaluation of Normalized Atrophy (SIENAx, part of FMRIB Software Library) [22]. Manual segmentation of the caudate nucleus, putamen, globus pallidus and thalamus was performed using Software for Neuro-Imaging Processing in Experimental Research (SNIPER), designed by the Laboratory for Clinical and Experimental Image Processing (LUMC, Radiology) [23]. 

### Statistical analysis

SPSS for Windows (release 16.0.) was used for data analysis. Group differences were analyzed with parametric or non-parametric tests when appropriate. To assess differences in demographics, UHDRS scores, brain volumes and basal ganglia hypointensities we used independent t-tests. Analysis of covariance was used to control group differences for possible confounding variables. Demographic and clinical characteristics and structural brain volumes have been published previously [23]. Pearson correlation analysis (r) was used to investigate associations of the amount of T2 hypointensities with UHDRS scores, basal ganglia volumes and CAG repeat length. The level of statistical significance was set at p≤ 0.01. Values of 0.01<p≤0.05 were considered as a trend toward significance.

## Results

There were no significant differences between groups for sex, age, years of education and UHDRS motor, cognitive and behavioral functioning (Table 1).  

 **Table 1. Clinical characteristics of the participants**

**Table:** 

	Carriers (n = 16)	Non-carriers (n = 13)
Male/ female^a^	6/10	5/8
Age in years	41.9 (10.0)	48.1 (9.0)
Education in years	12.9 (2.7)	12.5 (2.8)
CAG repeat length^b^	42 (40-49)	19 (16-24)
Probability of onset within 5 years^b^ (%)	17 (0-73)	
*UHDRS* Total motor score^c,d^ Verbal fluency SDMT Stroop color Stroop word Stroop interference Total behavioral score^d^	3.5 (0-10) 38.5 (13.6) 52.5 (11.8) 72.3 (13.0) 96.6 (10.6) 42.3 (7.7) 12.4 (11.4)	2.6 (0-6) 39.8 (10.5) 59.1 (10.5) 80.8 (16.9) 99.2 (20.0) 45.3 (6.9) 13.2 (15.2)

Values in the table are means (SD). No significant differences were found between carriers and non-carriers. Independent t-test analysis, ^a^Pearson's χ^2-^test, ^b^Median (range). ^c^Mean (range). ^d^Higher scores correspond with more abnormalities. UHDRS= Unified Huntington’s Disease Rating Scale. SDMT= Symbol Digit Modalities test. 

Caudate, putamen and globus pallidus volumes were smaller in carriers compared to non-carriers, also when corrected for total brain volume (Table 2) [23]. There was a trend towards a significantly increased amount of hypointensities in the basal ganglia of carriers compared to non-carriers (p= .02), also when corrected for age (p= .036) (Table 2) (Fig 2A and B). This difference was present for both the left (p= .022) and the right hemisphere (p= .017). Visual inspection of the hypointense pixels in each individual by two MRI specialists showed that hypointensities occurred almost exclusively in the globus pallidus in both carriers and non-carriers.

**Table 2. Basal ganglia volumes and T2-w hypointensities**

**Table:** 

	Gene carriers (n = 16)	Non gene carriers (n = 13)	P value
Caudate nucleus	6.4 (1.1)	7.4 (0.7)	.005**
Putamen	6.0 (1.1)	7.1 (1.0)	.009**
Globus pallidus	1.2 (0.5)	1.8 (0.5)	.006**
Thalamus	10.6 (1.3)	11.2 (1.0)	.173
Hypointensities^a,b^	396.8 (0-1503.5)	77.4 (15.76-249.3)	.02*

Values in the table are means with SD or range^a^ in parentheses. Student's t-test analysis, **p < 0.01, *p < 0.05. Volumes in cc. ^b^Number of hypointense pixels in the basal ganglia for the left and right hemisphere together.

**Figure 2. Basal ganglia hypointensities in two brain slices of one carrier (hypointensities: 957.8) (A); and one non-carrier (hypointensities: 77.2) (B)**

**A**

**Figure:**
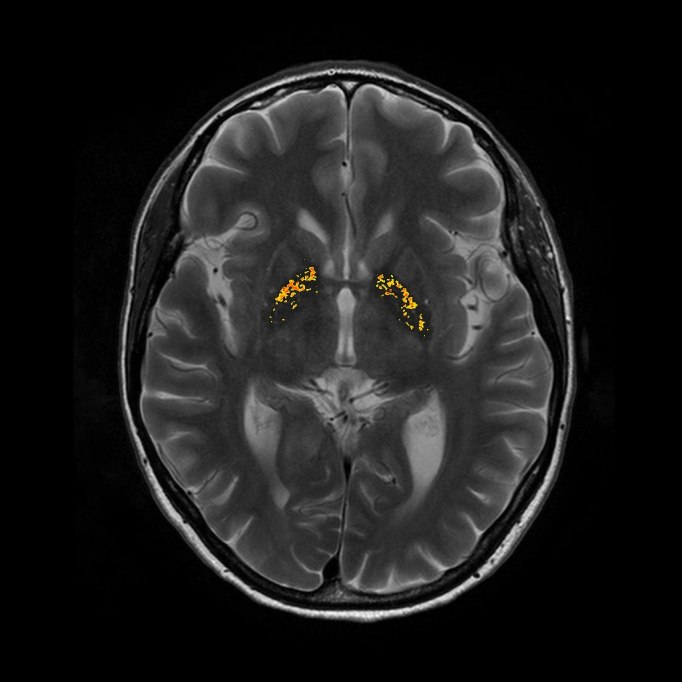

**Figure:**
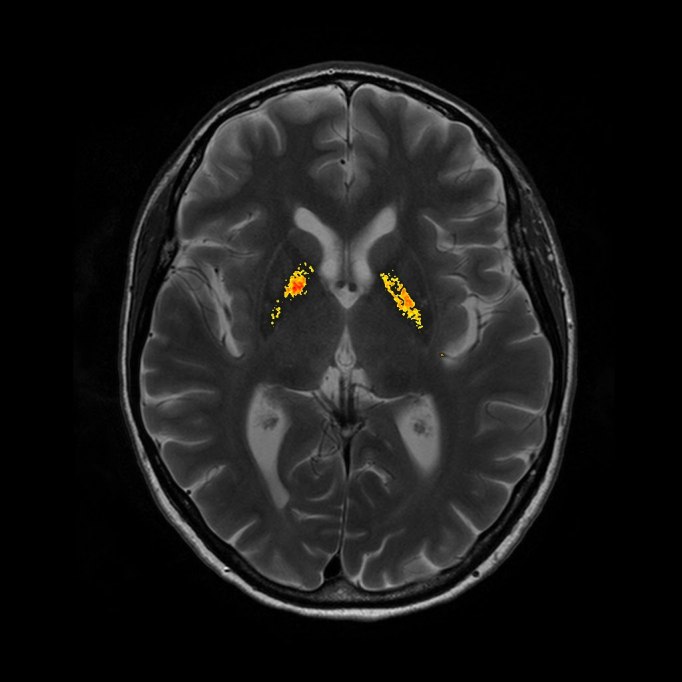

**B**

**Figure:**
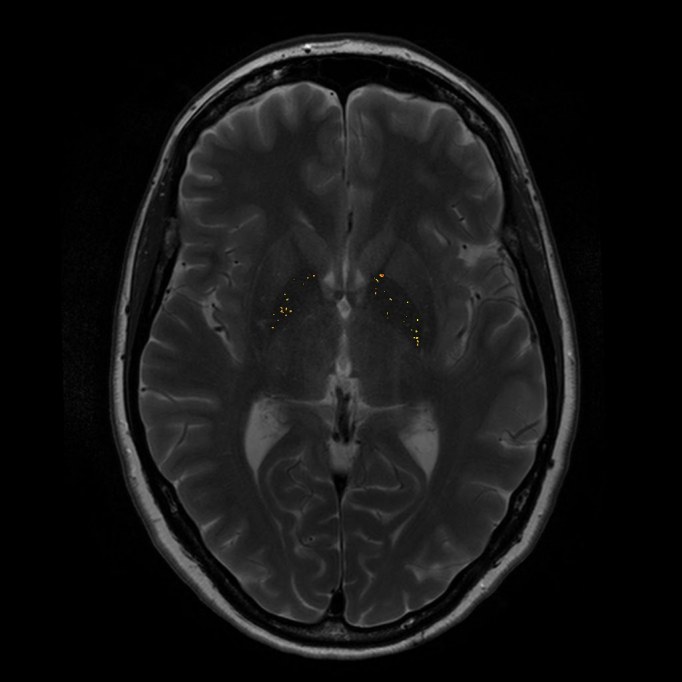

**Figure:**
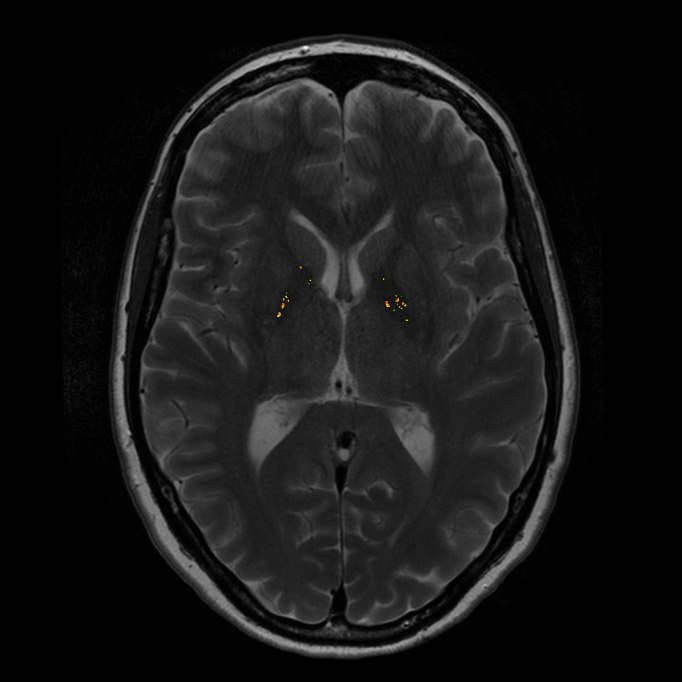

Significant associations between the amount of hypointensities and clinical or biological parameters were only found in carriers. An increased amount of hypointensities was associated with a higher score on the UHDRS motor scale, reflecting more motor abnormalities (r= .79, p< .001) (Fig 3A) and marginally with a lower score on the Symbol Digit Modalities Test (r= -.57, p= .028). Also, more hypointensities were strongly associated with a higher CAG repeat length (r= .84, p < .001) (Fig 3B) and a greater probability of developing symptoms within 5 years (r= .82, p< .001) (Fig 3C). A trend significant association was found between an increased amount of hypointensities and smaller globus pallidus (r=-.58, p= .02) and putamen volume (r-.53, p= .04). 

**Figure 3. Scatter plots of significant associations in carriers between an increased amount of hypointensities and higher UHDRS total motor score (r= .79) (A), longer CAG repeat length (r= .84) (B), and greater probability of symptom onset within 5 years (r= .82) (C)**

 **A**

**Figure:**
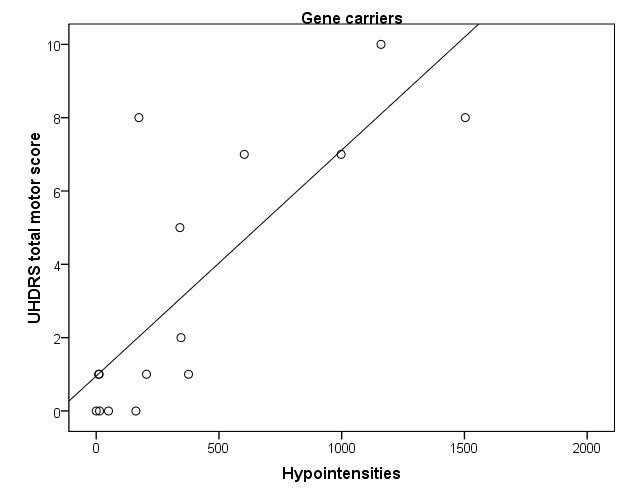

**B**

**Figure:**
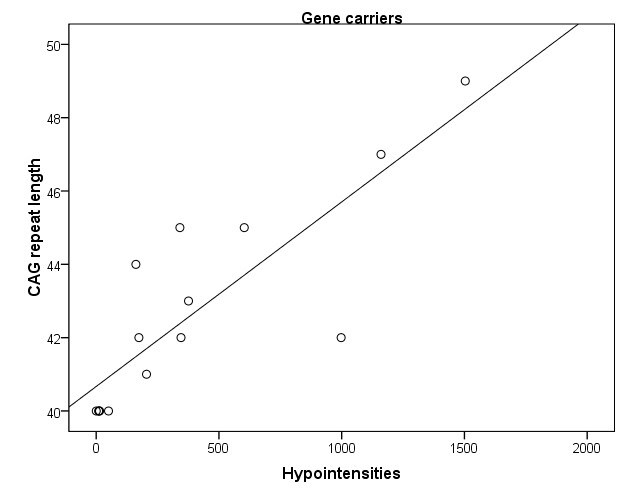

**C**

**Figure:**
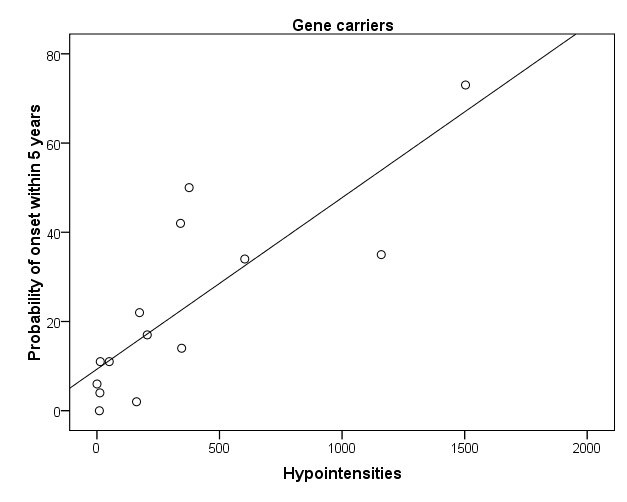


## Discussion

To our knowledge this explorative study is the first that demonstrated an increased amount of hypointensities on T2-w MRI in the basal ganglia of premanifest HD gene carriers. Furthermore we found a very strong association between the amount of hypointensities and clinical and biological HD characteristics.

Increased concentrations of iron have been suggested as the major cause of hypointensities on T2-w images [8] [11] [12] [13]. Iron plays an important role in normal neurophysiologic processes and is stored mainly in ferritin molecules. Increased iron levels are believed to lead to a process of oxidative stress and neuronal damage [24] [25]. In normal individuals, iron levels increase and subsequently T2 signal intensity decreases with age in the basal ganglia specifically [25] [26] [27]. Premature and excessive deposition of iron accumulates in the basal ganglia, and has been associated with neurodegenerative diseases, like AD and PD [2] [3]. Interestingly, the specifically damaged basal ganglia area in HD and the age at onset in midlife, whereas mutant huntingtin is expressed ubiquitously in the brain throughout development, may suppose a role for iron in the pathogenesis of HD [27]. Post-mortem studies indeed showed abnormal iron deposition in basal ganglia of HD patients [5] [7]. Furthermore two study groups demonstrated altered T2 relaxation times in basal ganglia of HD patients even early in the disease, and attributed this to pathological iron deposition [10] [14] [15] [16]. 

In our study we demonstrated that an increased amount of hypointensities on T2-w MRI is already present in the premanifest phase of HD. Furthermore we found that more hypointensities were strongly associated with a higher probability of approaching age at onset, confirmed by the association with more subtle motor abnormalities and slower psychomotor speed in these individuals. Elevated brain ferritin iron, as measured with field-dependent T2 relaxometry, has been related to the age at onset of AD and PD as well [27]. We also showed that an increased amount of hypointensities was associated with smaller volumes of putamen and globus pallidus, structures that were found to be altered in carriers compared to non-carriers. This strengthens the assumption that the observed increased amount of hypointensities represents a pathological phenomenon.

That hypointensities were almost exclusively found in the globus pallidus in both carriers and non-carriers is in accordance with the observation in normal individuals that increases in iron levels and decreases in T2 signal intensity in this structure are typically apparent already by the age of 30, while caudate and putamen remain isointense in middle adulthood [25]. Furthermore, Vymazal et al. demonstrated significant altered T2 shortening only in the globus pallidus of manifest HD gene carriers compared to controls [10]. We confirmed their finding that more hypointensities were associated with a longer CAG repeat length, suggesting that higher genetic disease load may be related to a higher content of toxic forms of iron [10]. 

To explain the premature iron deposition in HD carriers different hypotheses should be considered. In the basal ganglia iron bound to transferrin can transverse the blood-brain barrier by transferrin receptors in the vascular endothelial cells and is normally transported to other brain sites by means of axonal transport [3] [28]. HD related axonal damage and neuronal death may result in a decrease in this axonal transport and, with a continuing uptake, this could lead to the accumulation of iron in the basal ganglia [9] [10] [13]. Another hypothesis is that oligodendrocytes try to repair and remyelinate defects in the basal ganglia caused by mutant huntingtin. Oligodendrocytes have the highest iron content of all brain cells, thus leading to increased basal ganglia iron levels [16]. Furthermore, it is possible that breakdown of neuronal materials, like myelin, may release iron which is then translocated to the basal ganglia [16]. Another explanation is that huntingtin plays a role in iron homeostasis, since it was found to be required for normal regulation of the transferrin receptor, and it was modulated with it in response to iron need. Altered iron regulation could then lead to increased iron uptake and consequently brain iron levels [7] [29] [30]. Whether iron accumulation is the consequence of a neurodegenerative process or its primary cause, it can be argued that reducing brain iron levels as well as reducing oxidative stress could be an important therapeutic intervention [14]. 

The main limitation of the present study is that T2 hypointensities in HD have not yet been correlated directly with pathologic findings [6]. Also, there is no consensus on the most desirable MRI method for measuring brain iron. Many pulse sequences have been used to detect the presence of iron in tissues in vivo [8] [10] [14] [31]. Therefore, it is necessary to compare our T2-w hypointensity quantification with other methods. 

The strength of our MRI method is the fully automatic, quantitative and easy to administer processing of brain images to detect pixel-based T2-w hypointensity using high field imaging. Furthermore, the associations found with clinical and biological parameters emphasize the relevance of an increased amount of hypointensities in basal ganglia of premanifest HD. Whether the amount of hypointensities increases in HD development and is related to evolving structural brain damage and clinical manifestation of HD will be investigated in a follow-up study of the current cohort and in a new larger sample, including manifest HD carriers.

In conclusion an increased amount of hypointensities on T2-w MRI can be observed in basal ganglia of premanifest carriers of the HD gene and may reflect excessive iron deposition and a role for iron in the neuropathology of HD. Furthermore this phenomenon is associated with clinical and biological disease characteristics and might serve as a biomarker for HD. 

## Acknowledgements

The authors thank dr. Y.A.M. Grimbergen for her help in motor assessments and L. van de Wiel for her help in assessing the protocols. 

## Funding information

There were no funding sources.

## Competing interests

The authors have declared that no competing interests exist.
